# Radical treatment of Sister Mary-Joseph nodule: case report and literature review

**DOI:** 10.11604/pamj.2021.40.161.28407

**Published:** 2021-11-16

**Authors:** Amine Majdoubi, Tarek Bouhout, Marouan Harhar, Achraf Mirry, Serji Badr, Tijani El Harroudi

**Affiliations:** 1 Surgical Oncology, Faculty of Medicine of Oujda, Mohammed VI University Hospital, Regional Oncology Center, Oujda, Morroco,; 2 Pathology Department, Mohammed VI University Hospital, Oujda, Morroco

**Keywords:** Umbilical skin metastasis, stages of the tumor, Sister Mary-Joseph nodule, radical surgery, case report

## Abstract

Sister Mary-Joseph nodule (NSMJ) is a cutaneous metastasis of the umbilicus, rare and accounts for 2-3% of the patients with advanced stages of colorectal adenocarcinoma. Here we report the observation of a 48-year-old Moroccan man, referred to our hospital to manage a painful ulcero-budding nodule of the umbilicus; computed tomography revealed that the processes infiltrated the urachus and the bladder. Laboratory parameters were normal and radical surgery was performed to remove the tumor and embryological remnant of the umbilicus. The histological assessment confirmed the sigmoidal origin of the umbilical nodule. This kind of disease always poses a problem of treatment. It was considered for a long time as an outdated stage of tumor disease that deserves just palliative treatment. Several cases published in the international literature with radical treatment had good survival and evolution, which gives hope to patients with this disease.

## Introduction

Sister Mary-Joseph nodule (NSMJ) is an umbilical skin metastasis that manifests as a firm, ulcerated and painful mass. Rare in intra-abdominal cancers of digestive or gynecological origin [1], associated with the poor prognosis, with a survival time average of two months without treatment, and poses a therapeutic problem because it has long been considered an outdated stage of the disease and requires palliative treatment. Here, we report the observation of a 48-year-old man who referred to our training for surgical management of an umbilical budding that changed character by becoming aggressive, he has received a surgical treatment that carries the umbilical mass, the sigmoid, and resection of bladder flange with end-to-end anastomosis of the colon, and suture of the slice of the bladder section, the histological assessment revealed the NSMJ originated from sigmoid adenocarcinoma. Through a review of recent literature, we will discuss the usefulness of aggressive surgery associated with adjuvant chemotherapy to treat this disease, we also specify its epidemiological, diagnostic, and therapeutic aspects.

## Patient and observation

A 48-year-old man from Moroccan East presented to the outpatient department with a six-month history of an umbilical mass, generalized body weakness, and abdominal pain. He was operated on (one year and six months ago) appendicular peritonitis with a suppurated appendix in the local hospital of Taourirt. He also was on oral anti-diabetic medication (metformin 1000 mg 1cp3*/day) for two months at the time of presentation.

**Figure 1:**
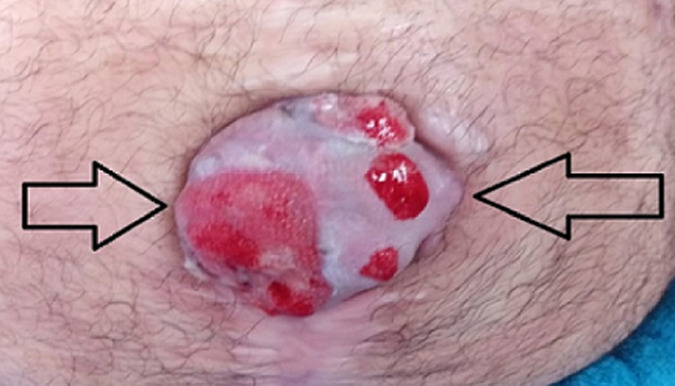
Sister Mary-Joseph nodule aspect on the admission

**Clinical findings:** on admission, we found on physical examination, an ulcero-budding mass (Figure 1), which appeared ten months after the treatment of peritonitis is which manifested in the beginning as a hard mass of 3 cm without inflammatory signs insight and which changed character after three months becoming fixed to the deep planes and measuring six cm, with inflammatory signs. Examination of the other systems was unremarkable, the patient had no Bowel obstruction, or rectal syndrome, drug history or family history of tumor, or any symptoms suggestive of colic manifestations.

**Figure 2:**
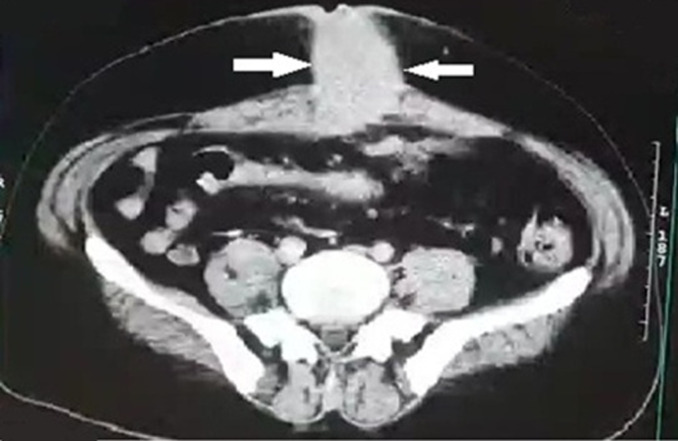
CT-scan of the abdomen; this image shows an umbilical mass

**Diagnostic assessment:** laboratory parameters were normal, two computed tomography were performed out at six months intervals, the first at the beginning of the symptomatology objectified the presence of a urachal cyst, while the second revealed the existence of an infiltrating process of the sea urchin. A skin biopsy was performed, returning in favor of the granulation tissue. An abdominal-pelvic computed tomography (Figure 2) was performed, objectifying the presence of: a urachal-cyst that manifested like an oblong structure, extending from the umbilicus to the dome of the urinary bladder measuring 68 * 18 mm. Two incisional-hernias, with mixed intestinal and mesenteric content: the first, median, measuring 61 mm, and the seconds, right lateral with a collar measuring 32 mm.

**Therapeutic intervention:** the patient was hospitalized, diagnosis and management were discussed with the patient. He benefited from an exploratory laparotomy under general anesthesia. We found that it infiltrated the sigmoid colon (it was perforated), the umbilical wall, and the bladder. This finding justified the resection of the umbilical mass, segmental resection of the sigmoid, resection of bladder flange with end-to-end anastomosis of the colon, and suture of the slice of the bladder section.

**Figure 3:**
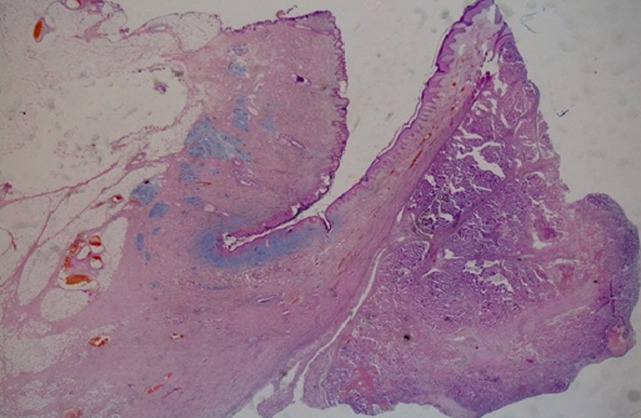
microphotography at low magnification shows the presence of a carcinomatous proliferation in the dermis, (HE; 10X)

**Figure 4:**
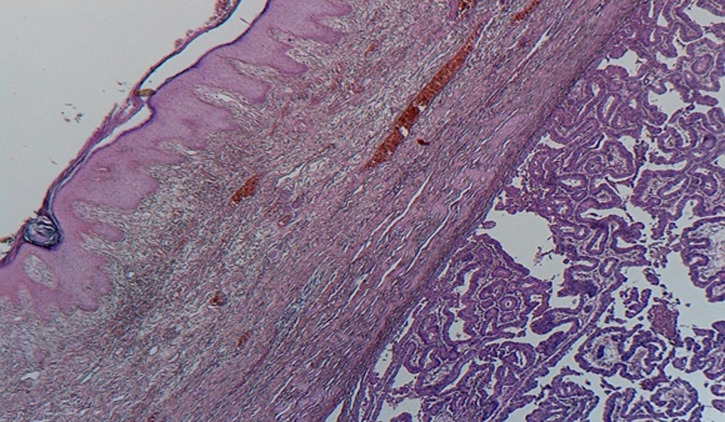
microphotography shows that the carcinomatous proliferation in the dermis is identical to those found in the colonic tumor, (HE; 40X)

**Figure 5:**
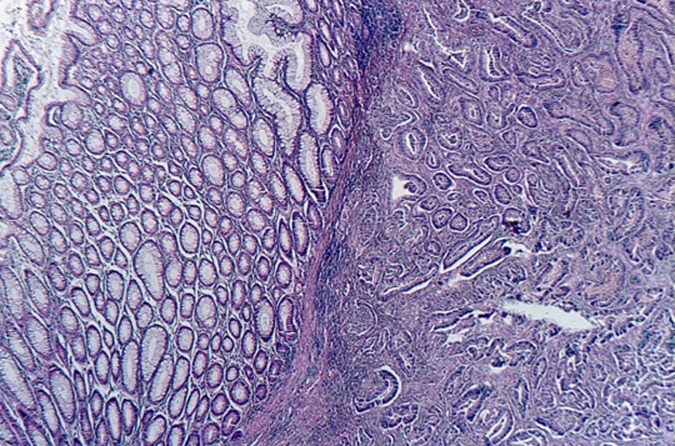
microphotography of the colonic tumor, showing infiltration of the colonic wall by a well-differentiated adenocarcinoma, (HE; 100X)

The histological assessments (Figure 3, Figure 4, Figure 5) of the resectioned parts retained the diagnosis of NSMJ with: for the bladder: no tumor proliferation, chronic inflammatory reshaping of the bladder mucosa; for segmental colectomy of the sigmoid: a well-differentiated and infiltrating adenocarcinoma of 2 * 2.5 * 4 cm, exceeding the sub-serous with a perforated wall, the limits of peripheral surgical resection are healthy (>4 cm), absence of vascular emboli or peri-nerve infiltration, lymph node curage 13 N-/13 N, tumor classified Pt4N0Mx; for the umbilical resection: skin localization of a well-differentiated adenocarcinoma. The tumor was located 6 mm from the deep resection boundary 4 mm from the nearest lateral resection boundary. Other lateral resection limits are healthy (more than 1 cm from it).

**Follow-up and outcomes:** postoperative follow-up was simple. During a staff of a multidisciplinary consultation meeting, the size of the tumor, the negativity of the extension assessments indicate adjuvant chemotherapy based on XELOX (oxaliplatin and capecitabine) for six months. The patient is regularly followed in our training (we opted for clinical, biological, and radiological monitoring by three months after a decline of 18 months, no recurrence detected).

**Patient perspective:** during the time he was hospitalized and after the treatment, the patient was delighted with the care she received and was optimistic about the outcome of her condition.

**Informed consent:** the patient was informed about the case report, why her case was peculiar and the author's interest in publishing her case. She willingly gave informed consent to allow the authors to use her photos for this case report.

**Patient's consent:** informed consent was obtained from the patient for us to use the pictures.

## Discussion

The sister Mary-Joseph nodule (NSMJ) is a rare skin metastasis localized at umbilicus skin, observed in the advanced stages of tumor disease of abdominopelvic origin [1]. Since its first description in 1949 by Sir Hamilton Bailey, in his manuscript Physical Signs In Clinical Surgery, the eponym describing the umbilical nodules pays tribute to his surgical assistant at St Mary Hospital “Sister Mary-Joseph”; she had noticed that some patients were following intra-abdominal tumors with umbilical nodules [2]. An umbilical nodule can be benign or malignant. Pseudo nodules of sister Mary-Joseph include all the umbilical lesions of the benign origins (polyp, endometriosis in 32% of cases, keloid scar). It represents about 57% of all umbilical lesions. Primary lesions (melanoma, basal cell carcinoma, and adenocarcinoma) represent just 20% of umbilical nodules [3]. In secondary cases (about 80% of malignant umbilical nodules), described in 52% for digestive cancers (stomach, gallbladder, pancreas, hail, colon and rectum), 28% gynecological origin (ovary, uterus), 10% urinary origin or unknown origin in 15 to 30% [3]. For men, gastric origin is the most common cause. For women, the ovarian location is incriminated first [4].

The NSMJ concerns 2 to 6% of colorectal adenocarcinomas [2], to the best of our knowledge, just 300 cases of NSMJ were published in the literature since 1960, and most of them were from gastrointestinal or gynecological tumor origins. In Morocco, the sigmoid origin was never reported, and we are the first to have published it. For colic cancers, skin metastases occur after a period of latency of two to three years after primary tumor surgery [5], typically at the site of the abdominal scar next to the umbilicus [5,6]. The NSMJ development mechanism is poorly known. It´s explained by the diffusion of a tumor through the lymphatic or venous and arterial network of the umbilicus, the extension by continuity from the anterior surface of the peritoneum or around the embryological remnant of the umbilicus (the falciform ligament, median umbilical ligament, or remnants of the vitelline duct) [3].

Clinically, the NSMJ manifests as a firm, ulcerated, painful, and irregular mass, with purulent or bloody discharge. Its color can be white or bluish violet or brownish-red, the size is usually less than 5 mm, but nodules larger than 6 cm have been reported in the literature [7]. The contribution of imaging is crucial. It makes it possible to identify the primitive and to study tumor extension in case of a locally advanced tumor. It also allows us to discuss differential diagnoses (pseudo-nodules of Sister Mary-Joseph) [7]. The diagnosis of certainty is purely histological. Usually, a biopsy or fine-needle aspiration makes the diagnosis [8]. It shows tumor infiltration of the skin. Most often, it is gastric adenocarcinoma in men and ovary in women [7]. SMJN is a worrying sign associated with the advanced stage of tumors, with a survival time average of two months without treatment, 11 months with it, and only 13.5 percent of patients living after two years, regardless of the etiology of the primary tumor [9].

The therapeutic approach depends on the clinical condition of the patient and the local status of the tumor. For inoperable patients or with locally evolved tumors, palliative chemotherapy always finds its indication; it improves overall survival from 34 weeks up to 3 years from diagnosis, with an average of 10 months [7,9,10]. Palliative surgery is possibly limited to colostomies or tumor reduction surgery. Laparoscopy should always be preferred [10,11]. For operable patients (in the good general state) when curative surgery is possible, especially if the umbilicus is the only metastatic site. Hemicolectomy and resection of the umbilical mass with the embryological remnant of the umbilicus improves overall survival (21 months) and reduces the risk of recurrence [10,11]. In the literature (Table 1), adjuvant chemotherapy combined with surgery improves survival and allows a decrease in local recurrences, facing chemotherapy alone [10-15].

**Table 1:** published cases with the aggressive approach of treatment

Reference	Age and sex	Colonic segment	Metastatic site	Neo-adjuvant chemotherapy	Adjuvant chemotherapy	Type of surgery	Recurrence	Treatment of recurrence	Overall survival
Y. Iwata *et al*.	42 female	The ascending colon	Umbilicus and peritoneal nodule	12 months	FOLFOX	Right hemicoloectomy; excision of umbilical metastatic cancer	Four months after surgery	Chemo +surgery (partial colectomy including anastomosis, bilateral oophorectomy, and the resection of the peritoneal nodule)	50 months
J-S. Chen *et al*.	60	Adenocarcinoma (ADK) the ascending colon	Umbilicus	No		Right hemi colectomy and excision of umbilical metastatic cancer	FOLFOX	No	_
Grossi U *et al*.	60 male	ADK sigmoide	Umbilicus	Bevacizumab for 9 months		Sigmoid resection and greater omentectomy the umbilicus and the entire urachal ligament up to the bladder dome	1 year	Chemotherapy	48 months
Wu YY *et al*.	37 male	Caecum	Inguinal lymph node + umbilicus	Refused by the patient		Right hemi colectomy and resection of the umbilical mass were performed through a median abdominal incision; excision of the enlarged lymph nodes of the right groin was performed for biopsy			4 months
Our patient	48 male	Sigmoid	Umbilicus Infiltrate the bladder	No	FOLFOX	Sigmoid resection and the umbilicus and the entire urachal ligament up to the bladder dome	No	None	Still alive

## Conclusion

The NSMJ is a worrying sign associated with an advanced tumor for a long time that deserves palliative treatment, with an average survival of 11 months for colic origin despite well-conducted chemotherapy. Although the NSMJ reflects tumor aggressiveness, the international literature shows that radical surgery (resection on the bloc of the tumor, embryological remnants of the umbilicus, and resection of umbilicus skin) improves survival when combined with adjuvant chemotherapy.
